# Dendrimers Show Promise for siRNA and microRNA Therapeutics

**DOI:** 10.3390/pharmaceutics10030126

**Published:** 2018-08-08

**Authors:** Volha Dzmitruk, Evgeny Apartsin, Aliaksei Ihnatsyeu-Kachan, Viktar Abashkin, Dzmitry Shcharbin, Maria Bryszewska

**Affiliations:** 1 Institute of Biophysics and Cell Engineering of NASB, 220072 Minsk, Belarus; alexei.ihn.k@gmail.com (A.I.-K.); viktar.abashkin@gmail.com (V.A.); d.shcharbin@gmail.com (D.S.); 2 Institute of Chemical Biology and Fundamental Medicine SB RAS, 630090 Novosibirsk, Russia; eapartsin@mail.ru; 3 Center for Theragnosis, Biomedical Research Institute, Korea Institute of Science and Technology (KIST), 02972 Seoul, Korea; 4 Department of General Biophysics, Faculty of Biology and Environmental Protection, University of Lodz, 90-236 Lodz, Poland

**Keywords:** dendrimer, denderiplex, dendron, miRNA, siRNA, microRNA, antimiR, gene therapy, gene delivery, gene drugs

## Abstract

The lack of an appropriate intracellular delivery system for therapeutic nucleic acids (TNAs) is a major problem in molecular biology, biotechnology, and medicine. A relatively new class of highly symmetrical hyperbranched polymers, called dendrimers, shows promise for transporting small TNAs into both cells and target tissues. Dendrimers have intrinsic advantages for this purpose: their physico-chemical and biological properties can be controlled during synthesis, and they are able to transport large numbers of TNA molecules that can specifically suppress the expression of single or multiple targeted genes. Numerous chemical modifications of dendrimers extend the biocompatibility of synthetic materials and allow targeted vectors to be designed for particular therapeutic purposes. This review summarizes the latest experimental data and trends in the medical application of various types of dendrimers and dendrimer-based nanoconstructions as delivery systems for short small interfering RNAs (siRNAs) and microRNAs at the cell and organism levels. It provides an overview of the structural features of dendrimers, indicating their advantages over other types of TNA transporters.

## 1. Introduction

The targeted regulation of expression of therapeutically relevant genes using various types of nucleic acids (NAs) has been demonstrated in numerous in vitro and in vivo biological model systems [1,2,3]. Genes can be regulated by means of plasmid DNA or small RNA molecules. The delivery of exogenous genes encased in DNA plasmids to the cell nucleus requires a number of biological barriers to be overcome [4]. Thus, multicomponent delivery systems such as viral particles or CRISPR-Cas9 are applied to facilitate plasmid transfer into target nuclei [5].

Recent achievements in genetic research have provided many new tools for manipulating gene expression, each possessing its own individual advantage. For example, the natural mechanism of gene expression regulation by RNA interference (RNAi) [6], discovered during the 1990s, has fewer biological barriers to gene silencing than DNA plasmids because it takes place exclusively in the cell cytoplasm at the post-transcriptional level [7]. Small interfering RNAs (siRNAs) and microRNAs (miRNAs) are two major types of RNAi effectors. The nucleotide sequence of the effector chain determines the target gene to be regulated. In essence, RNA interference works by blocking expression of the targeted gene by arresting or destroying the messenger RNA via the native cell protein complex (RNA-inducedsilencing complex, RISC) bound to short regulatory RNAs that act as guides. This mechanism is described in more detail in a series of reviews [6,7,8,9].

RNA interference as a gene therapeutic tool provides the advantage of a much broader choice of target proteins than traditional treatment approaches [10]. Generally, the action of low molecular mass drugs on target proteins is based on an “enzyme–substrate” type of interaction. This leaves a wide range of pathogenic proteins unreachable by traditional drugs owing to the conformational limitations on binding to the targeted protein-active site, the low enzymatic activity of the pathogenic protein, the variability and mutations of the target protein structure, resistance to access by small-molecule drugs [11,12], and other factors [13]. Since the target protein to be silenced by RNAi is determined by the sequence of short microRNAs and siRNAs, these would seem to be efficient tools for potentially inhibiting the expression of any protein by specifically arresting the mRNA before the protein of interest is translated. MicroRNAs and siRNAs, as RNA interference effectors, are therapeutically interesting because of their ability to overcome the major limitations of traditional low molecular weight drugs.

Large numbers of therapeutics associated with siRNA and microRNA action are currently in the process of preclinical and clinical trials [2,14,15,16]. However, the absence of economically affordable, effective, and low-toxicity transporters of TNAs remains a major problem in the clinical application of siRNAs and microRNAs in the gene therapy concept [17].

Nowadays, all vectors for gene delivery can be divided into two large principal classes: viral and non-viral (synthetic). Recombinant retroviruses, adeno-associated viruses, and lentiviruses, etc. serve as viral vectors [18]. These were the viral vector base for drugs first approved for clinical application and thus was the first gene therapy on the pharmacological market [19]. Nowadays, a number of gene therapeutic drugs are available. Glybera was the first approved for use in the European Union in 2012 [19], after Strimvelis was approved in 2016 in European Union and Imlygic was approved in 2016 in both the European Union and the USA [20]. Viral vectors are highly effective but they have some weaknesses and limitations for use as medications [18]. The difficulties associated with large-scale production of these drugs result in high prices, making them unaffordable for many patients: for example, from 2012 to 2017, only one patient was able to receive Glybera [20].

Therefore, the development of new materials capable of binding, transporting, and releasing TNAs is high on the scientific agenda. The scope of the current review covers the design of synthetic delivery systems for small therapeutic RNAs based on dendrimer-related polymeric materials: dendrimers and/or dendrons (structural elements of dendrimers), describing their efficiency in in vitro and in vivo models The description and comparison of dendrimer-based delivery vehicles with other non-viral vectors can be found in other reviews [21,22,23]. 

## 2. Dendrimers

**Figure 1:**
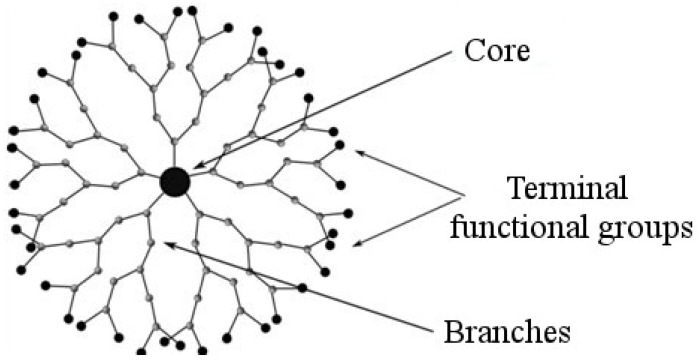
Schematic structure of a dendrimer.

Dendrimers seem to be promising platforms for developing non-viral RNA delivery systems. They are highly symmetrical hyperbranched polymers with tree-like structures; their branches of monomeric units diverge in all directions from a central core [24]. All dendrimers share general similar structure: they consist of a central core and more than two branches of the length depending on dendrimer generation. The number of surface sites on each branch is doubled with every generation increase starting from the number of core binding points (Figure 1). A property that distinguishes dendrimers from other classes of macromolecular compounds is the so-called dendritic effect [25]. This can consist in: (1) the distinction between the properties of dendrimers and their linear analogues (multivalent effect); or (2) altering the intensity of any of the properties (biocompatibility, binding efficiency to transported molecules, transfection efficiency, etc.) with increasing generation (generation effect). If there is no structural regularity in linear and branched polymers because of uncontrolled polymerization then the polymer generation cannot be determined. It is mostly the generation effect that determines the advantage of dendrimers over other polymer classes in biological applications, so it is important to choose the appropriate generation as well as the dendrimer’s chemical structure to solve any particular research problem [26]. The extent of the dendritic effect can be affected by the nature and size of the dendrimer core and the degree of cooperativity among the terminal functional groups. 

Dendrimers are attractive carriers for small RNAs owing to a combination of properties that distinguish them from other polymer classes. Their main advantage is monodispersity (all dendrimer molecules are identical and have a well-defined size after synthesis) [27,28], in contrast to other polymers, which are the products of uncontrolled polymerization. Another of their features is the large number of functional terminal groups, which enables them to bind many molecules to their periphery. Complexes of dendrimers with biological molecules, by analogy with lipoplexes and polyplexes, are called dendriplexes [28]. The option of choosing the monomeric units and their structures enables scientists to design dendrimers with particular physico-chemical characteristics, to select the appropriate molecular weight and the density of functional groups within branches and on the periphery, and to link different targeting groups to them [29]. Moreover, dendriplexes can circulate in the body for a long time [30]. All these advantages show dendrimers to be a promising platform for gene therapy delivery. 

**Figure 2:**
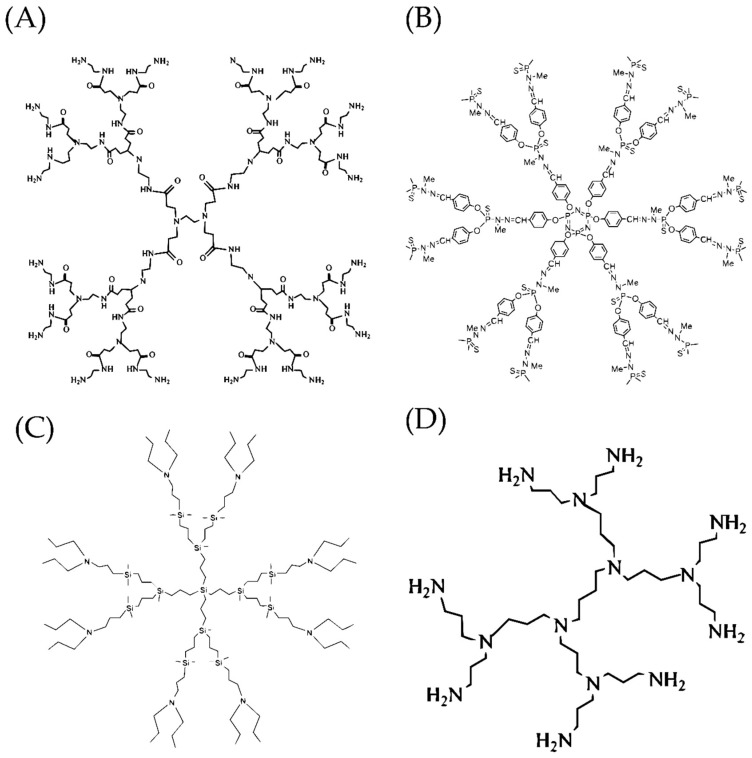
General structures of cationic polyamidoamine (**A**), phosphorous (**B**), carbosilane (**C**) and polypropylenimine (**D**) dendrimers, most commonly used to deliver therapeutic nucleic acids. Dendrimers of the second generation are shown.

A wide range of dendrimers has been synthesized to date [31,32], but only five cationic classes—polyamidoamine (PAMAM), polypropylenimine (PPI), carbosilane (CBS), poly-l-lysine (PLL) and phosphorus dendrimers—are mainly used for delivery of siRNAs and microRNAs (Figure 2). 

The common property that shares dendrimers of any kind is rapid increase of molecular density towards surface groups. The more core multiplicity, the shorter and more rigid monomer units the denser dendrimer’s structure. Thus, any kind of dendrimers have their own generation limits due to steric limitation of the terminal groups. For example, the highest generation ever synthetized was obtained for phosphorus dendrimer G12, as they are built up of lengthy monomers [33].

### 2.1. Polyamidoamine Dendrimers (PAMAMs)

PAMAMs were historically the first class of dendrimers to be synthesized [24]. They have been used in a wide range of applications owing to their simplicity of synthesis and commercial availability. Various linear (e.g., ethylenediamine (EDA) [34], diaminobutane (DAB)) or branched (triethanolamine, TEA [35]) amines and artificial compounds (e.g., polyphenylene-vinylinene [36]) can serve as the core of PAMAM. The branches are constructed from aliphatic aminoamides, the nitrogen atom acting as the branch point. Primary amino groups are often used as classical terminal groups, capable of binding nucleic acids efficiently [37]. The peripheries of PAMAM are also available for decoration with various functional fragments (e.g., polyethylene glycol (PEG), fatty acids, peptides) [38,39] to modulate the dendrimer’s bioavailability and functions. Nanoconstructions based on PAMAM dendrimers or their branches are of great scientific interest as they allow the properties of different synthetic and natural biomolecules to be combined to create a perfect RNA delivery system [40].

### 2.2. Polypropylenimine Dendrimers (PPIs)

PPI dendrimers contain a butylene diamine core and propylene imine monomers. Two types of imines can be present in a PPI dendrimer structure: tertiary, located in the nucleus and monomers; and primary, located at the periphery [41,42]. The primary and tertiary amino groups are completely protonated under physiological conditions, ensuring the ability of the dendrimers to bind negatively-charged therapeutic NAs [43,44]. The high charge density at the PPI periphery allows them to transfer a large number of therapeutic molecules into a cell simultaneously. PPI dendrimers, like PAMAM dendrimers, are commercially available.

### 2.3. Carbosilane Dendrimers (CBS)

Carbosilane dendrimers usually possess a C-Si backbone and their bifunctional methylsilane fragments act as the bifurcation/branching points of monomeric unit branches. Several techniques for synthesizing carbosilane dendrimers with a compact tetrafunctional cores (e.g., tetraallylsilane, tetravinylsilane) [45,46] and a more voluminous trifunctional nucleus (trioxybenzene) [47] have been described. Since the core is relatively small, dendrimers of this class have a high density of functional groups at the periphery. Carbosilane dendrimers are significantly less toxic than other classes [48,49]. Nevertheless, they have a facility for penetrating into cells of different origin as well as neurons [50,51]. It has been shown that carbosilane dendrimers and their complexes can overcome the blood–brain barrier [52].

### 2.4. Polylysine Dendrimers (PLL Dendrimers)

PLL dendrimers are hyperbranched monodisperse analogues of linear oligopeptides widely used for transfection and for drug and gene delivery [53]. They are built from α-l-lysine fragments linked by amide bonds. The lysine residues thus constitute the branches and act as branch points. PLL dendrimers are excellent examples of combining the properties of linear biocompatible polymers with the advantages of the dendritic effect. The main advantage of polylysine dendrimers is their easy biodegradation under the action of proteases and, in consequence, low cytotoxicity [54,55,56,57].

### 2.5. Phosphorus-Containing Dendrimers

Phosphorus-containing dendrimers generally consist of a thiophosphate or cyclotriphosphazene core and extended branches containing stable aromatic thiophosphohydrazones [58,59]. Such a structure allows steric limitations to be overcome during synthesis: phosphorus dendrimers can be grown up to generation 12 [60], in contrast to PAMAM dendrimers for which the maximum generation number is 10 [61]. Also, because of their specific structure, phosphorus dendrimers possess voluminous internal cavities in which small molecules can be encapsulated [60]. On the other hand, the extended branches make the density of functional groups on the periphery of the dendrimer comparatively low. Phosphorus dendrimers have proved effective as transfection agents: they easily penetrate through the cell membrane and promote cell transfection with plasmids even in the presence of bovine proteins [62,63].

## 3. The Mechanism of Action of Dendriplexes in a Cell

Dendrimers bearing cationic groups inside and outside their structures can bind negatively charged nucleic acids effectively, mainly by electrostatic interactions, thus forming complexes called dendriplexes by analogy with polyplexes [64,65,66]. The excess of positive charges on a dendriplex surface promotes penetration through cell membranes, generally by adsorption endocytosis. Generally, endocytosis can be effected by various mechanisms: phagocytosis, adsorption endocytosis, pinocytosis, or clathrin- and caveolin-mediated endocytosis [67,68,69]. The mechanism of cell uptake of dendriplexes depends significantly on their size and can involve one or more of the mechanisms mentioned [70]. After being taken up by cell, they appear in endosomes and remain there for some time until the endosome is disrupted. Cationic dendrimers prevent acidification of endosomes before they mature into late endosome or lysosome form by buffering the contents (proton sponge effect). The secondary and tertiary amines of dendrimers become protonated as the pH in the endosome decreases, and this is followed by rupture of the endosomal membrane (umbrella effect) [71]. These processes terminate in endosomal escape, dendriplex disintegration and release of the components of the complex into the cytosol, where the biological effects of RNAi occur. Dendriplex dissociation with the release of nucleic acids occurs either spontaneously during dendrimer swelling [72] or by competitive binding to polyanions, for example with blood glycosaminoglycans [73]. The detailed mechanism of endosomal escape of dendriplexes and their subsequent dissociation is still debated and is apparently individual to each type of dendrimer.

During the past two decades tremendous progress has been made in the dendrimer-based delivery of genetic material and therapeutic molecules. Nevertheless, the toxicity of cationic polymeric macromolecules remains an acute problem. Cationic dendrimers, as a rule, have protonated amino groups on their periphery. Unbound charged groups interact with negatively charged cell membranes, mainly electrostatically. As a result, this interaction leads to membrane thinning, formation of pores and damage to membrane integrity, which in turn leads to necrotic cell death [74]. Such nonspecific interactions are responsible for the cytotoxic side-effects of cationic dendrimers in biological systems. On the other hand, nonspecific electrostatic interactions also occur between dendrimers and serum proteins [75,76], thus significantly reducing the in vivo toxicity [77]. To improve the biocompatibility of cationic dendrimers, two basic strategies are usually applied: (1) the inclusion of biodegradable components—core or branching units, e.g., unstable or degradable by cellular metabolites [78]; and (2) partial neutralization of the surface cationic groups by PEGylation, addition of oligopeptides, oligosaccharides, acyl chains, etc. [74]. Partial degradation of the branches [79] or the use of dendrons instead of the complete spherical structure of the dendrimer significantly reduces the cationic surface charge density, which has a beneficial effect in reducing the cytotoxicity of TNA delivery systems [80].

The scope of this review is to summarize experimental data on the use of dendrimers for delivering short therapeutic RNAs in vitro and in vivo. Papers that are mostly concerned with the possibility of using dendrimers to deliver siRNAs that inhibit the expression of non-therapeutic targets, such as a green fluorescent protein or luciferase, are not reviewed here [81]. Descriptions of dendrimers and various dendrimer-based constructs are reviewed, along with their efficacy in delivering siRNAs, microRNAs mimics, and microRNA antagonists to inhibit therapeutically important genes. Special attention is paid to one of the most promising applications in modern medicine: the combined effect of chemotherapeutic drugs and dendriplexes to suppress the growth of cancer cells.

## 4. PAMAM + siRNAs Dendriplexes

Small interfering RNAs (also known as short or silencing RNA) are exogenous double-stranded RNAs of 21–23 base pairs in length. They can mimic the functions of natural regulators of gene expression (endogenous microRNAs) without undergoing shortening by the Dicer enzyme and can be directly embedded in the RNA-induced silencing complex (RISC) [8]. Most of the scientific data on dendrimer-based siRNA delivery refers to PAMAMs and their various modifications. 

Despite of some disadvantages, unmodified dendrimers are effective as carriers of RNA interference regulators. Cationic TEA-PAMAM G5 can deliver siRNA precursors Dicer-substrate siRNAs,(dsiRNA) for multitarget suppression of the human immunodeficiency virus (HIV). Zhou et al. achieved a more than a twofold decrease in the expression of p24 (one of the key viral proteins) in human lymphocytes in vitro. In vivo studies on HIV-infected humanized mice models yielded even more impressive results: injections of TEA-PAMAM G5 complexes with a cocktail of 4 dsiRNA (dsiTAT, dsiREV, dsiCD4 and dsiTNPO3) into some mice led to undetectable viral particle levels accompanied by a decrease in the total CD4+ lymphocyte count [35]. Otherwise, reduction in the CD4+ T-cell count is a serious medical complication requiring certain isolation procedures before and after radiotherapy. Nevertheless, this approach represents a much more targeted and therefore more sparing therapeutic regimen for HIV treatment than traditional antiretroviral chemotherapy.

**Figure 3:**
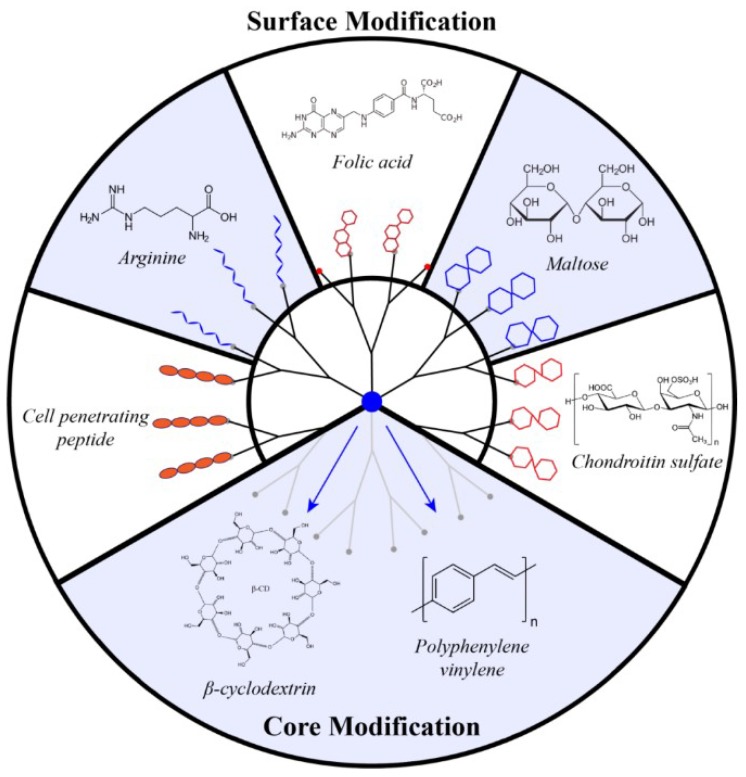
Chemical modifications of polyamidoamine (PAMAM) dendrimers to improve dendrimer-based delivery of small interfering RNAs (siRNAs).

The overwhelming majority of studies on siRNA delivery involve modified PAMAM dendrimers. Owing to the commercial availability of these dendrimers, scientists have plenty of options to increase their biocompatibility, reduce their toxicity, and improve targeted delivery by numerous chemical modifications (Figure 3). A detailed description of the nanocomposites studied and their biological effect in delivering siRNA is summarized in Table 1.

### 4.1. PAMAM Surface Decoration with Amino Acids and Peptides

Peng et al. conducted a series of studies to identify the most efficient PAMAM dendrimer modification as a delivery vehicle for siRNA siHsp27 [39,82,83,84,85]. The siRNA was designed to suppress heat shock protein Hsp27, which is essential for drug resistance in prostate cancer. Substitution of terminal amine groups in TEA-PAMAM G4 by arginine residues before siRNA binding [82] or addition of the peptide Arg-Gly-Asp-Cys to already-formed (TEA- PAMAMG5) dsiRNA complexes [83] results in a rapid increase of intracellular siRNA/dsiRNA penetration in vitro since the dendriplexes acquire the properties of cell-penetrating peptides (CPP). However, in vivo tests on murine xenografts revealed almost no significant difference in Hsp27 expression level or tumor size after treatment with modified and unmodified PAMAM-based complexes. The most likely reason is the capacity of cationic dendrimers to penetrate into target cells in vivo either with or without oligopeptides.

Small RNAs are able to bind to not only dendrimer surfaces but also to inside branch sites if internal amines became protonated, as shown by internally quaternized PAMAM dendrimers with acetylated surface aminogroups (QPAMAM-NHAc,s). Transfer of binding points from the surface to internal cavities allows the terminal cationic charge to be decreased by substitution with hydrophobic groups, and this is followed by a significant reduction of general side-effect toxicity [87]. Following addition of luteinizing hormone-releasing hormone (LHRH) peptides to such dendriplexes leads to targeted absorption by cancer cells and suppression of programmed protein expression [88,103]. The delivery of siBcl-2 by LHRH-QPAMAM reduces Bcl-2 expression in cancer cells by more than by 75% [87,88,103] and promotes accumulation in tumor and liver tissues in vivo [88].

The above-mentioned compositions combine the advantages of dendrimers (binding and release of siRNA, stability in biological fluids, etc.) and CPP (targeted and efficient cell penetration). One of the key factors in dendrimer-based siRNA carrier design is the number of oligopeptides per dendrimer molecule. Since cell-penetrating peptides usually possess a positive charge, their combination in high concentrations with cationic PAMAM dendrimers results in an overcharge, which in turn can lead to increased toxic side-effects and reduced delivery efficiency both in vitro and in vivo [90].

### 4.2. Surface Decoration with Oligosaccharide

One widespread method for neutralizing dendrimer-associated toxicity is partial substitution of cationic groups with various di- or oligo-saccharides [104]. In general, such modification significantly increases biocompatibility on the one hand, but on the other hand it lowers the efficiency of binding, delivery and release of nucleic acids [91]. The use of such modification motifs in vivo could overcome such pharmacokinetic obstacles as untimely early siRNA release from complexes in the bloodstream rather than a target cell. Thus, such compounds are not deemed suitable for therapy, but can be efficient for prevention. It was shown that an siRNA against NF-κB p65 can be delivered into liver cells by maltose-modified PAMAMG3 with cyclodextrin, with subsequent protection from hepatitis infection in vivo [92].

Another way to improve dendrimer-based delivery is to make the dendrimer amphipathic by adding ‘lipid tail’-like alkyl chains. Spherical dendrimers carrying long linear hydrocarbon chains on the surface can form analogy of micelles, like lipids, called dendrisomes [34,86]. The length of the hydrocarbon chain affects the ability to form dendrisomes [84,105] as well as the time of siRNA release in vivo [34]. Lipid-like dendrimers compared to commercial Lipofectamin2000 were shown to be more efficient in Bcl-2 silencing by the corresponding siRNA impact [86].

### 4.3. Core Modification

Replacement of the ethylenediamine dendrimer core with highly branched rigid structures (e.g., polyphenylenevinylenes or β-cyclodextrin) allows more biocompatible dendrimers to be created. The rigid polyphenylenevinylene core in combination with the flexible PAMAM branches results in a highly effective siRNA carrier for neurological treatment. It induces a high level (>90%) of siRNA penetration into neurons with subsequent protection of the cells from apoptosis [36] and autophagy [95] even if rat neuron death is exogenously-induced by *N*-methyl-d-aspartate. A similar construction based on the β-cyclodextrin core and PAMAM G3 dendrons showed accelerated wound healing in rats with diabetes: injected dendriplexes carrying metalloproteinase-9 siRNA suppressed the relevant protein expression in vivo [93]. A branched core leads to a decrease in surface charge density and an increase in internal cavity space. Some believe that these factors make it possible to obtain less-toxic constructs without reducing their effectiveness in the delivery of siRNA.

### 4.4. Amphiphilic Lipid-Like Dendrons

**Figure 4:**
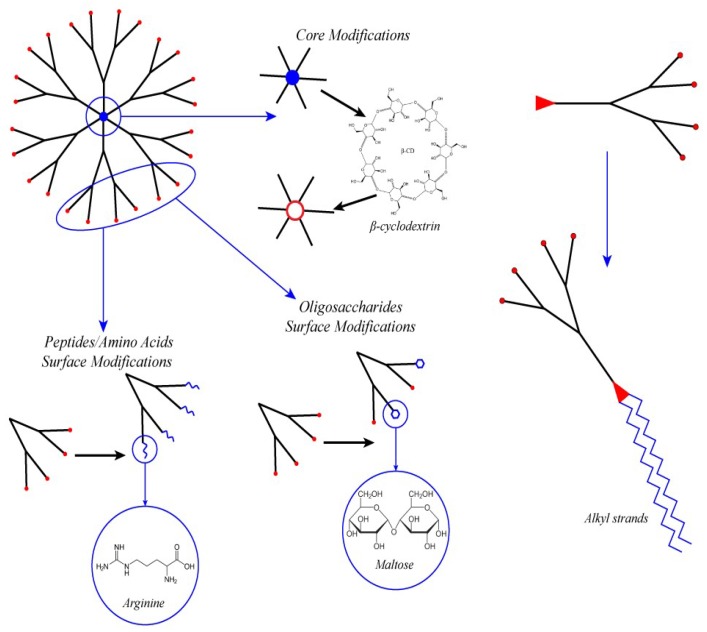
Dendron modifications to create lipid-like small RNA transporters.

An interesting approach is to combine the advantages of dendrimers with the amphiphilic properties of lipids (Figure 4). The most appropriate structure for that purpose is not the whole dendrimer but its separate branches. To obtain amphiphilic dendrimeric molecules, one [39,84] or two [85,96] alkyl strands of different lengths [84,105] are attached to the dendron’s focal point. Such lipid-like structures can form dendrisomes spontaneously just as lipids build up into micelles. They effectively deliver small RNAs via the dendrisome surface and decrease the expression of the targeted proteins Bcl-2 and CD4 both in vitro and in vivo with no significant cytotoxicity. Surface decoration with arginine residue allows the degree of penetration to be increased as amphiphilic dendrons also achieve the properties of cell-penetrating peptides [39].

## 5. Non-PAMAM + siRNAs Dendriplexes

There have been significantly fewer studies recruiting other types of dendrimers for siRNAs delivery. They are represented by several groups of cationic dendrimers: poly-l-lysine [97,98] and carbosilane dendrimers [48,49,50,99,101,102], and phosphorus-containing dendrimers [48,49,100].

Carbosilane dendrimers were designed and synthesized by F. Javier de la Mata and Rafael Gómez and their colleagues. NN16 dendrimers possess easily hydrolysable Si-O bonds in their internal architecture and diimine groups on the periphery and seem to have an optimal balance between efficiency and cytotoxicity among naked dendrimer carriers for delivering short-chain RNAs. The NN16 dendrimer of the second generation makes complexes with oligonucleotides up to 200 nm in size because it is significantly smaller than third- and fourth-generation PAMAM dendrimers [102,106,107]. This small size enables it to penetrate into a wide range of small cells, for example cells of the immune system. Here, the instability of the branches ensures effective intracellular release of the “load” and makes them non-toxic to cells [50,101]. Such dendrimers have proved effective in delivering a siRNA that suppresses the vital HIV-1 proteins COX2, p24, NEF, and GAG, reducing the viral load from 35 to 60% [50,99,101,102] in vitro in different cell lines. A similar study on the suppression of HIV-1 in primary human peripheral blood mononuclear cells (PBMCs) was performed with a fourth-generation phosphorus dendrimer. The authors noted more pronounced toxicity but significantly greater effectiveness in virus growth repression. The phosphorus dendrimer complexed with anti-NEF siRNA in vitro showed little toxicity [100]. Earlier in our studies we conducted a comparative analysis of the effectiveness of three major dendrimer groups for siRNAs binding and transfer into cancer cells. On the one hand, carbosilane dendrimers exhibited significantly lower cytotoxic effects than phosphorus and PAMAM dendrimers. On the other hand, their intracellular internalization was much less effective. Phosphorus and PAMAM dendrimers can not only facilitate intracellular siRNA penetration, but also exert additional influence on cancer cells, causing necrosis, apoptosis, and autophagy and thus enhancing the anti-cancer therapeutic effect [48,49].

## 6. Dendriplexes with microRNA Mimics and Antagonists

MicroRNA (miRNA) and siRNA are both short non-coding nucleic acids that regulate the synthesis of proteins in numerous cell types via RNA interference mechanism [108].These molecules have precursors, longer RNA strands or short hairpin RNAs, that are shortened by the intracellular enzyme Dicer to their functional length of 18–25 base pairs [9,109]. However, the fundamental differences between these two RNAi effectors are important. The first difference lies in their biogenesis. SiRNAs are synthesized exogenously, while microRNAs are endogenous and are the products of non-coding RNA in introns [110]. The structures of functional units also differ: the mature microRNA is single-stranded, while siRNA is double-stranded. However, the key and principal difference is the number of mRNA targets affected by these two RNA types. As a rule, siRNA has one mRNA target with which it has a perfect complementary interaction. A microRNA has a number of mRNA binding sites, possibly hundreds, because it can interact with mRNAs with complementarity mismatches [111]. This imperfect complementary makes microRNAs able to regulate the expression of several proteins simultaneously. At the final stage of gene silencing, microRNAs can cause not only complete degradation of mRNA, but also repression of the target mRNA and/or inhibition of reading with or without subsequent degradation of the mRNA [110]. 

MicroRNAs can control metabolic, pathological, and homeostatic processes in cells by both gene downregulation and upregulation [112,113]. For example, the expression of the regulatory microRNA profile is altered when a normal cell is transformed into a cancer cell. The expression of oncogenic microRNA can increase, leading to metastasis and tumor proliferation (upregulating), or the synthesis of regulatory proteins that cause the death of cancer cells can be suppressed (downregulating) [114]. The therapeutic approach based on the use of microRNA involves two different strategies:

1. Similar to siRNA, the synthetic mimics of microRNAs (miRs) or their precursors can be introduced into the cell with subsequent target gene silencing triggered by RNA interference.
2. Otherwise “malignant” endogenous microRNAs in the cells can be arrested by synthetic oligonucleotides called microRNA antagonists (antimiRs). These oligonucleotides form strong duplexes with microRNAs and block their activity [10].

During the past 10 years there have been numerous studies on microRNA action. These studies have developed from recent discoveries of (1) dysregulation of microRNA expression profiles in pathological cells (creation of microRNAs and their target libraries); and (2) the capacity of microRNAs to affect cell phenotypes after altering the expression of several target genes simultaneously [10]. Many microRNA sequences were discovered during a short time period, so any newly reported microRNA was given a number. Thus, microRNAs are commonly named according to a pattern exemplified by “hsa-miR-8204b”. Here, “hsa” denotes *Homo sapiens* origin, “mir” refers to a precursor and “miR” to a mature microRNA, the number (8204) is a unique identifier (microRNAs with lower id numbers were discovered earlier), and the letter after the number indicates there are two or more mature microRNA with very similar sequences. There are more rules for microRNA nomenclature relating to other parameters, which are used in many databases [115,116]. 

As the libraries grow, unique microRNA expression profiles could provide a basis for personalized therapy and a tool for effective monitoring [114]. Some new therapeutic approaches based on microRNA mimics and antagonists have reached the phase II clinical trials [117]. 

Although much fundamental knowledge about microRNAs has been acquired, their therapeutic use has been severely limited by lack of an appropriate delivery system; microRNA mimics and siRNAs encounter several biological and physiological barriers once they are inside the organism. Most preclinical studies on dendrimer-based miR delivery (Table 2) have been aimed at suppressing malignant tumors [117]. Lack of microRNA regulators (miR-34a, miR-93 and miR-200c) in a dormant osteosarcoma led to a dramatic change in its phenotype from avascular to rapidly-progressing angiogenic. Restoring the levels of these miRs in the osteosarcoma Saos-2 and MG-63 cell lines more than doubled the dormant period of the osteosarcoma cells both in vitro and in vivo. To restore the endogenous downregulated levels of miR-34a, miR-93 and miR-200c, Satchi-Fainaro and colleagues used an aminated polyglycerol dendrimer as carrier for their synthetic mimics. The latent stage of the osteosarcoma (and the remission stage) was prolonged, widening the window for chemotherapy [118]. One of the main targets of miR-34a in osteosarcoma cells is the proto-oncogene MET (cMET) [118], while in pancreatic cancer cells (MiaPaCa-2) it targets procaspase-3 and Bcl-2 [119]. These proteins protect tumor cells from the initiation of apoptosis. Silencing of procaspase-3 and Bcl-2 expression by (chondroitin sulfate)-PAMAM-miR-34a complexes led to an increase in the apoptotic fraction of cancer cells (22% vs. 6% in controls). The efficiencies of microRNA delivery by the PAMAM dendrimer, chondroitin sulfate-functionalized PAMAM, and commercial lipofectamine2000 were almost identical in vitro [119].

The mimic of miR-7 microRNA was studied as a human glioma growth inhibitor in vitro and in vivo; miR-7 regulates the expression of several target proteins simultaneously, including the tumor growth and apoptosis regulators EGFR, PI3K, and AKT-2, in human glioma U251 cells. PAMAM dendrimers with conjugated folic acid residues delivered miR-7 into the U251 cells efficiently in vitro with subsequent significant silencing of EGFR, PI3K, and AKT-2. The ability of the FA-PAMAM conjugate to overcome the blood–brain barrier, to transfect cells effectively, and to be selectively accumulated in cancer cells resulted in significant inhibition of tumor growth in vivo and prolongation of survival time in mice xenografted with gliomas [38].

Zhou et al. synthesized 1500 different modular dendrimers consisting of a polyamide core, carbosilane branches with hydrolysable bonds, and various thiol-containing compounds on the surface [121]. Such a large-scale screening allowed scientists to identify a delivery system for let-7g microRNA with an optimal balance of low hepatotoxicity, selective accumulation in the liver, and high efficiency in inhibiting the growth of aggressive tumors in mice in vivo. MicroRNA let-7 is downregulated in many tumor types and belongs to the tumor suppressor family. The biodegradable modular 5A2-SC8 carrier proved the best, and its application to miR delivery in vivo in mice with aggressive hepatocellular carcinoma resulted in a 13-fold increase of let-7g expression in the liver after i.v. injections, with no significant hepatotoxicity and a pronounced survival benefit [121]. 

The only study that did not concern cancer treatment was published by Gray et al. [120]. They had designed two hybrid compounds to increase the biocompatibility of dendrimers: amphiphilic Janus-type-PAMAM dendrimers consisting of a cationic dendron G3 at one side of the focal point and a peptide (a penetrating CR9 or targeting CGGRGDS) at the other side of the focal point. The efficacy of amphiphilic dendrimers was demonstrated in endothelial cells upon delivery of a miR-126 mimic. As a result, the mRNA level of the target SPRED1 (a signal protein) was halved, which led to a significant improvement in cell proliferation and integration. These results could form a basis for miR-based therapy for myocardial and ischemic diseases by promoting angiogenesis in affected regions [120].

The second microRNA-based therapeutic strategy is intracellular introduction of antimiRs. This approach does not require an obligatory delivery system. MicroRNA antagonists are single-stranded oligonucleotides complementary to mature upregulated endogenous miR. Since their mechanism of action is not associated with recognition by intracellular proteins, various chemical modifications of oligonucleotides are widely used: 2’-O-methylation, locked nucleic acids (LNA), unlocked nucleic acids (UNAs), etc. Such modifications protect antimiRs from recognition by endogenous nucleases and binding to immune system proteins [10,122], but they are still able to block target microRNAs. Thus, the lifespan and circulation time in the body of modified antimiRs are significantly longer than for unmodified ones. For example, an antimiR-122 with a LNA modification is already undergoing clinical trials as a drug against hepatitis C [16]. Nevertheless, intracellular penetration and targeted uptake to develop new generations of effective gene therapy drugs for a wide range of diseases remain problematic.

Concerning the second strategy, only a few recent studies have been published dealing with delivery of the antimiR-21 microRNA antagonist in combination with anti-cancer chemotherapeutics. This is discussed below.

## 7. Combined Effect of siRNA and microRNA with Therapeutic Drugs

The distinctive hyperbranched structure of dendrimers enables them to incorporate low molecular mass chemotherapeutics into their internal cavities and simultaneously bind functional nucleic acids to their surfaces (Table 3). Thus, it is possible to strike cancer cells with a combination of “weapons” targeted at multiple proteins or at the different steps in their expression. The Akt protein is important for evading apoptotic cell death in ovarian cancer cells. Thus, suppression of Akt expression enhances the ability of paclitaxel to combat cancer cells in vivo and in vitro. The use of a TEA-PAMAM G6 dendrimer as an siRNA carrier made the synergistic effect of the dendriplex and chemotherapeutics 85% greater in vivo than the action of either factor alone. This marked enhancement was achieved by a combination of intratumoral injections of the siRNA-dendrimer complexes and traditional intravenous injection of paclitaxel [123].

Cause–effect relationships between resistance to cancer chemotherapy and alterations in microRNA expression profiles have recently been established. In the “dendriplex + drugs” approach, low molecular mass drugs are delivered and released inside the cell simultaneously with siRNA/microRNA; this allows drug resistance to be blocked and at the same time enhances the effect of the corresponding chemotherapeutics. Moreover, the incorporated chemotherapeutics could have significantly increased solubility, enhanced intracellular penetration, and improved targeting, leading in consequence to a notable reduction of dosage, which in turn significantly reduces the severity of side effects.

As for prostate cancer chemotherapy, resistance can be weakened by knocking down the MAPK protein (mitogen-activated protein kinase) in the MAPK/ERK signaling pathway, which has a significant role in prostate carcinogenesis. Thus, inhibition of p42-MAPK expression potentiates the antitumor effect of the anti-diabetic drug metformin [124]. Although the most effective PAMAM dendrimers for siRNA transfection are between generations G4 and G7 [133], Monteagudo and co-authors demonstrated the ability of the smallest PAMAM G1 to reduce the expression level of the target protein by 80% in prostate cancer cells in vitro [124]. They demonstrated key evidence of enhancement of the chemotherapeutic effect when the immortality of the cancer cells was suppressed by the RNA interference machinery, using dendrimers as siRNA carriers.

Drug resistance can be overcome by targeting the major vault protein (MVP) in breast cancer cells. MVP hampers the entry and persistence of doxorubicin (DOX) molecules in tumor cell nuclei where DOX acts, and thus causes DOX resistance. The combined delivery of MVP-siRNA and DOX by PAMAM G5 functionalized by hyaluronic acid resulted in a significant increase in cytotoxicity in vitro. The authors reported that delivery within dendriplexes enhanced tumor targeting, increased blood circulation time, and lowered in vivo toxicity in comparison to DOX alone. A synergetic action is also achieved through increased accumulation of DOX in cancer cells both in vitro and in vivo when it is delivered by the dendrimer via an enhanced permeability and retention (EPR) effect along with the receptor-specific interaction between the hyaluronic acid residues with CD44 receptors that are hyperexpressed on the tumor cells [125].

In contrast to siRNA and microRNA, antimiRs are not involved in the cell machinery so intracellular enzymes do not recognize them. Thus, in vivo application of antimiRs does not require a delivery system, since their chemical modification enables them to circulate long in the body with no loss of functional activity. That is why few articles on dendrimer-based delivery of antimiRs can be found. 

One of the newest therapeutic concepts is the application of antimiRs to block the microRNAs responsible for chemotherapy resistance (e.g., let-7, miR-34, miR-451, miR-200 [117]). A combination of increased cancer cell chemosensitivity and simultaneous local exposure to the chemotherapeutics can overcome the drug resistance. This effect was demonstrated in a series of studies of the joint delivery of various chemotherapeutics and antimiR-21 complexed by PAMAM G5 dendrimer in vitro. MiR-21 is overexpressed in the cells of at least nine different solid tumors [130], and its arrest triggers apoptosis in breast cancer [129,130], glioma [131,132], and glioblastoma [127,128] cells. In these studies, the chemosensitivities of cancer cells of different etiologies to 5-fluoroutorouracil [127,129], Taxol (paclitaxel) [128,130], and temozolide [131,132] were significantly increased as a result of heterotarget delivery. This effect was achieved owing to enhanced cell uptake of antimiR-21 (71.3% vs. 2.1% in the control) as well as the chemotherapeutics [127].

## 8. Conclusions

Only 20 years passed after the discovery of RNA interference as a gene silencing tool [134] before the first therapeutic drugs based on RNAi reached phase III clinical studies [135]. The discovery of applications of RNA interference in medicine opened wide opportunities for scientists to create a completely new class of drugs, the targets of which can potentially be any protein. Moreover, this tool allows disturbed cell regulation to be normalized, involving both gene downregulation and upregulation. Despite rapid progress in RNAi research, a number of problems related to toxicity, targeting, biological barriers, and delivery remain unsolved. For local superficial therapeutic application, for example in ophthalmic diseases, chemical modifications of siRNA can help to avoid such problems since these nucleic acids are stable enough to remain effective and thus are currently undergoing clinical trials [136]. However, in order to develop the full potential of RNA interference in therapy for incurable and severe diseases, the problem of packaging and delivery in vivo must be solved.

Along with viral, liposomal, and polycationic particles, dendrimers are now being actively investigated as carriers for small RNAs. A unique tree-like compact structure distinguishes dendrimers from other polymers. The combination of numerous active sites for binding with the presence of internal cavities, and flexibility in the synthesis and modification of dendrimers, led to the development of two distinctive therapeutic concepts: (1) chemical modifications of cationic dendrimers that allow their toxicity to be significantly reduced while maintaining delivery efficiency; and (2) the combined delivery of chemotherapeutic drugs inside the cavities and small RNAs at the periphery, which has shown particular effectiveness against chemoresistant cancer cells of various etiologies.

The current review has highlighted preclinical studies on microRNA- and siRNA-containing constructs developed against cancers, HIV, neurodegenerative diseases, diabetes, and hepatitis. Thus, delivery systems based on dendrimers could potentially become a universal therapeutic approach. The key factor in the functional difference of such constructs is the small RNA sequence, which determines its specific intracellular target. The possibility of selecting any target protein for siRNA, along with microRNA targets, offers great potential for the development of personalized medicine using dendrimers.

## Figures and Tables

**Table 1:** Dendrimer-based gene delivery systems for siRNA therapeutics. TEA: triethanolamine; HIV: human immunodeficiency virus; dsiRNA: Dicer substrate siRNA; EDA: ethylenediamine; HSP: heat shock protein; LHRH: luteinizing hormone-releasing hormone; PEG: polyethylene glycol; PLL: poly-l-lysine; PMBC: peripheral blood mononuclear cell.

Target Protein/Type of Short RNA	Object	Dendrimer/Dendrimer Based Construction	Effect	Ref.
**PAMAM dendrimers**				
Hsp27/siRNA	Human prostate cancer cells (PC-3)	Arginine-terminated TEA-PAMAM G4 (G4Arg) dendrimer	80% reduction of Hsp27-mRNA, Hsp27 protein expression dropped by 85%	[82]
	PC-3 prostate cancer xenografts in nude mice		Hsp27 protein expression decreased by 55%	
	Human prostate cancer cells (PC-3)	Complex of TEA-PAMAM G5/siRNA/oligopeptide E16G6RGDK	Hsp27 mRNA reduction by 60%, decrease of Hsp27 protein expression by 85%, reduction of cell viability by 55%	[83]
	PC-3 prostate cancer xenografts in nude mice		Hsp27 expression decrease by 70%, 5-fold inhibition of tumor growth	
	Human prostate cancer cells (PC-3)	Amphiphilic TEA-PAMAM dendrons G1,2,3, bearing C18 alkyl chain in focal point	Target mRNA decrease of 75%, protein expression decrease of 80%, 2.7-fold increase of apoptotic cells	[84]
	PC-3 prostate cancer xenografts in nude mice		Decrease of target mRNA by 50%, decrease of Hsp27 protein expression by 50%	
	Human prostate cancer cells (PC-3)	Arginine-decorated TEA-PAMAM Dendron G3, bearing an alkyl chain in the focal point	Decrease of target mRNA by 80%	[39]
		Amphiphilic Janus-type PAMAM G2 dendron bearing two alkyl chains in focal point	Decrease of Hsp27 mRNA by 80%, decrease of Hsp27 protein expression by 95%	[85]
	PC-3 prostate cancer xenografts in nude mice		Decrease of hsp27 mRNA by 60%, decrease of Hsp27 protein expression by 75%, 2.5-fold inhibition of tumor growth in vivo	
Cocktail of viral (HIV) Tat and Rev, lymphocytic CD4/TNPO3/dsiRNAs	T-cells and primary human PBMC	TEA-PAMAM G5 dendrimer	Decrease of viral p24 expression by >50% and CD4 expression by 60–75%	[35]
	HIV-infected humanized Rag2−/−γc−/− mouse model		Decrement of viral load up to 0%, prevent CD4+ T-cell level fall	
Bcl-2 (inhibitor of apoptosis)/siRNA	Human cervical adenocarcinoma cells (HeLa)	Dodecylated PAMAM G4 bearing 23 chains of C12	Decrease of target mRNA by 90%, protein Bcl-2 expression inhibition by 40%	[86]
	Human ovarian carcinoma cells (A2780)	QPAMAM-NHAc, internally quaternized and surface-acetylated PAMAM G4 modified with LHRH at the periphery	Inhibition of target mRNA by 85%	[87,88]
		Triblock PAMAM-PEG-PLL nanocarrier	Inhibition of target mRNA by up to 80%	[89]
Alpha-fetoprotein (AFP)/siRNA	C57BL/6 mice, hepatocarcinoma model	EDA-PAMAM G1 substituted by alkyl chains on the periphery	Selective accumulation in hepatocytes, decrease of target protein expression by 50% (C12) and by 90% (C15)	[34]
Multiple drug resistance protein 1 (MDR1)/siRNA	MDR1-positive mouse embryonic fibroblast (NIH 3T3) cells	Tat-Conjugated EDA-PAMAM G5 dendrimers	Target protein (MDR1) expression decreased by 35%	[90]
Transthyretin—transport protein (TTR)/siRNA	Hepato-carcinoma cells (HepG2)	EDA-PAMAM G2 decorated with glucuronylglucosyl-β-cyclodextrin	Decrease of target mRNA level by 60%	[91]
	Mice model BALB/c		Decrease of target protein (TTR) expression by 10%	
NF-κB p65- (regulator of inflammatory response)/siRNA	Rat alveolar macrophages (NR8383)	EDA-PAMAM G3- decorated with cyclodextrin and thioalkylated mannose fragments	Decrease of target NF-κB p65 mRNA level by 85%	[92]
	Mice model C57BL/6		Reduction of proinflammatory cytokines p65, TNF-α, IL-1β secretion by 75–85%	
MMP-9 (diabetic wound healing regulator)/siRNA	Rat fibroblasts (CRL1213)	PAMAM G3 with β- cyclodextrin core	Decrease of target MMP-9 mRNA level by 68%, decrease of target protein expression by 94%	[93]
	Sprague Dawley rats with induced diabetes		Enhancement wound healing (52% against 38% in control)	
Cocktail of Bcl-2, Bcl-xL, Mcl-1 (apoptosis inhibitors)/siRNAs	Human cervical adenocarcinoma cells (HeLa), human promyelocytic leukemia cells (HL-60)	PAMAM G3 and G4	Increased apoptotic cell fraction up to 30–40%	[48,49]
Angiotensin II receptor type 1 (AT1R)/siRNA	Cardiomyoblastic cells (H9C2)	EDA-PAMAM G4 dendrons conjugated with PEG-R9peptide	Reduction of protein AT1R expression by 60%	[94]
	Rats with induced ischemia		2.5-fold decrease of heart attack risk	
Cofilin-1 (regulator of neuronal death)/siRNA	Rat cerebellar granular neurons (CGNs)	TRANSGEDEN: Polyphenylenevinylene (PPV) core with flexible PAMAM branches	Knockdown of target mRNA by 85%, reduction of protein Ccofilin-1 expression by 80%	[36]
Beclin 1 (autophagy regulator)/siRNA	Rat brain rat neurons		Decrease of Beclin 1 mRNA by 90%, knockdown of Beclin 1 protein expression by 80%	[95]
TWIST1 (marker of breast cancer)/siRNA	Breast cancer cells (SUM1315)	YTZ3-15, TEA-PAMAM dendron G3 with two lipid tails in focal point	Decrease of TWIST1 mRNA and protein by 75–95%, reduction of epithelial-mesenchymal transition (EMT)-related (N-cadherin and vimentin) gene mRNA	[96]
CD4 (primary HIV receptor)/dsiRNA	Human hematopoietic CD34+ stem cells	Amphiphilic TEA-PAMAM dendron G3 bearing alkyl chain C18 in focal point, decorated with arginine	Decrease of CD-4 mRNA by 60%	[39]
	Acute lymphoblastic leukemia T-cells (CCRF-CEM)	Amphiphilic Janus-type- TEA-PAMAM dendrons bearing two alkyl chains	Decrease of CD4-mRNA by 55%, knockdown of CD4 protein expression by 80%	[85]
Cocktail of HIV-1 Tat/Rev (viral integrase)/dsiRNAs	PBMC CD4+, hematopoietic stem cells CD34+		Decrease of Tat/Rev mRNA level by 50–55%, inhibition of HIV replication in infected cells by 30–40%	
**Delivery of siRNA by non-PAMAM constructions**				
Cocktail Bcl-2, Bcl-xL, Mcl-1 (apoptosis inhibitors)/siRNA	Human cervical cancer cells (HeLa), human acute promyelocytic cells (HL-60)	PAMAM G3, G4; carbosilane G2; phosphorous G3, G4 (comparison study)	Apoptosis induction by cocktail of 3 siRNA: carbosilane (15–20%) < PAMAM (30–40%) < phosphorous G3 (45%) << phosphorous G4 (95%)	[48,49]
Apolipo-protein B (ApoB)/siRNA	Mice model C57BL/6	Poly-l-lysine G6 (KG6)	Decrease of mRNA in hepatocytes by 22% (aiApoBI) and by 50% (aiApoBII), low and very low density lipoprotein level in blood by 20–25%	[97]
PEPCK (glucose production regulator)/siRNA	Rat hepatocytes H4IIEC3	Combination of KG6 (dendritic poly(L-lysine) G6) and Endo-Porter peptide	Decrease of PEPCK-mRNA by 80%, knockdown of PEPCK protein expression by 95%, blood glucose level decrease by 70%	[98]
OCT1 (gluconeogenesis regulator via influence on metformin)/siRNA			Decrease of OCT1-mRNA by 80%, metformin (inhibitor of gluconeogenesis) action arrest	
Nef (necessary protein for HIV reproduction)/siRNA	CD4+-lymphocytes	Carbosilane (CBS) G2, G3 dendrimers	HIV-1 reproduction inhibition in vitro by 35% (G2) and by 50% (G3)	[99]
	PBMCs	Phosphorous G4 dendrimer	HIV-1 reproduction inhibition by 60%	[100]
COX2 (cyclooxygenase-2, stimulator of HIV propagation in brain)/pool of four siRNA sequences	Astroglioma cells (U87MG)	NN-16 G2 (carbosilane dendrimer)	Decrease of COX2 expression in HIV-infected cells to the level of uninfected cells	[101]
P24, NEF (HIV structural proteins)/siRNA			50% inhibition of HIV-1 propagation	[50]
P24, GAG1, NEF (HIV structural proteins)/cocktail of three siRNAs	T-cell lymphoma lymphoblasts (SupT1), primary PBMCs		35% inhibition of HIV-1 propagation	[102]
Bcl-2 (apoptosis inhibitor)/siRNA	Cell Line human ovarian carcinoma (A2780)	PPI G5-PEG-LHRH conjugate	Decrease of Bcl-2 mRNA level by 75%	[103]
	Human lung carcinoma (A549)		Decrease of Bcl-2 mRNA level by >95%	
	A549-derived lung carcinoma xenografts in a nude mouse model		LHRH conjugates increase accumulation of dendriplexes in tumor xenografts	

**Table 2:** Dendrimers for delivery of therapeutic microRNA mimics.

microRNA (Target)	Object	Dendrimer/Dendrimer Based Construction	Effect	Ref.
**microRNA Mimic Delivery**				
miR-7 (epidermal growth factor receptor)	Human glioblastoma cells U251	Conjugate of PAMAM folic acid (FA/PAMAM)	Decreased expression of proteins EGFR by 90%, PI3K by 50%, AKT-2 by 30%	[38]
	Immunodeficient mouse with induced glioma		Decreased expression of proteins EGFR by 50%, AKT-2 by 60%, reduction of tumor size	
duplex miR-126 (signal protein SPRED1)	Human umbilical vein endothelial cells (HUVECs)	Amphiphilic Janus-type-PAMAM dendrimer, consisting of dendron G3, bound to the penetrating peptide CR9 or targeting peptide CGGRGDS	Decrease of the level SPRED1-mRNA by 50%	[120]
let-7g (target is unknown)	Mice with induced aggressive hepatocarcinoma	Hybrid carbosilane dendrimer G2 with a polyamine core and thiol-containing surface groups	Inhibition of liver tumor growth in mice in vivo, let-7g expression was increased 13-fold in liver tissues after 48 h post intravenous (i.v.) injection	[121]
miR-34a (cMET, angiogenesis and tumori-genesis regulator), miR-93 (angiogenesis regulator factor HIF1α), miR-200c (prevents metastatic spread, pathway unknown)	Human osteosarcoma cells (Saos-2 and MG-63), SCID mice with Saos-2 derived tumors	Aminated polyglycerol dendrimer (dPG-NH2)	2–3 fold times increase in latent phase of osteosarcoma duration in vivo	[118]
miR-34a (procaspase-3 and Bcl-2)	Pancreatic cancer cells (MiaPaCa-2)	PAMAM dendrimer functionalized by chondroitin sulfate on the surface (CS-PAMAM)	Decreased viability of MiaPaCa-2 cells by 35%, 6.5-fold increase of the cell fraction in the apoptosis phase in vitro	[119]

EGFR: epidermal growth factor receptor.

**Table 3:** Combined delivery of therapeutic siRNAs and antagonists of microRNA with anti-cancer chemotherapy.

microRNA/siRNA (Its Target)	Object	Dendrimer/Dendrimer Based Construction	Effect	Ref.
**siRNA**				
p42 MAPK-siRNA (a protein of MAPK/ERK signaling cascade regulating transcription) + metformin	Prostate cancer cells (PCa)	EDA-PAMAM G1	Decrease of p42-mRNA by 85%, decrease of p42 protein expression by 70%, increased cells sensitivity to metformin	[124]
Akt-siRNA (ovarian cancer stimulator protein) + paclitaxel	Human ovarian carcinoma cells (SKOV-3)	TEA-PAMAM G6	Decrease of Akt-mRNA by 60%, decrease of the Akt protein expression by 40%, in cell viability decreased by 40% (dendriplex) and by 60% (dendriplex + paclitaxel)	[123]
	SKOV-3 xenograft nude mice model		Reduction of xenograft tumor size by 2 times (dendriplex) and by 4 times (dendriplex + paclitaxel)	
MVP-siRNA (major vault protein involved in breast cancer drug resistance) + doxorubicin (DOX)	Breast cancer cells (MCF-7/ADR)	EDA-PAMAM/hyaluronic acid conjugate	Significant knockdown of MVP protein expression, increased cytotoxicity of the dendriplex + DOX (IC_50_ = 11.3 μM) compared to DOX alone (IC_50_ = 48.5 μM)	[125]
	Xenograft of MCF-7/ADR in Nude BALB/c mice		Enhanced tumor target, higher intracellular accumulation, increased blood circulating time and reduced vitrotoxicity of DOX/denpriplex co-delivery compared to DOX alone	
Cocktail Bcl-2, Bcl-xL, Mcl-1 (apoptosis inhibitors) / siRNA + 5- fluorouracil	Human cervical cancer cells (HeLa)	Aminopiperidine-terminated phosphorus dendrimers G3 and G4	Synergistic effect of two anti-cancer agents (siRNA and chemodrug), enhancement of the apopotosis induction	[126]
**microRNA Antagonists**				
antimiR-21 + 5- fluorouracil	Glioblastoma cells (U251 and LN229)	TEA-PAMAM G5	Addition of dendriplex increase cell chemosensitivity to 5-fluorouracil	[127]
antimiR-21 + taxol			Decrease of miR-21 level by 90–95%, increase in cells chemosensitivity to taxol (IC_50_ = 60–160 nM)	[128]
antimiR-21 + 5- fluorouracil	Breast cancer cells (MCF7)		Increased chemosensitivity of cells to 5-fluorouracil, a prolonged cytotoxic effect	[129]
antimiR-21 + taxol			Decreased expression of p-AKT, Bcl-2, EGFR, STAT-3 proteins, increased sensitivity of cells to taxol	[130]
antimiR-21 + temozolomide	Glioma cells (U87)		Decrease of miR-21 level by 80–90%, an increase in the sensitivity of cells to temozolomide (IC_50_ = 7.5 μM)	[131]
antimiR-21 + temozolomide	Glioma cells (U251, LN229, U87)		Decreased expression of STAT-3 and p-STAT proteins, increased chemosensitivity of cells to temozolomide	[132]

## Abbreviations

TNA	therapeutic nucleic acid
RNAi	RNA interference
PAMAM	polyamidoamine
EDA	ethylenediamine
TEA	triethanolamine
PPI	polypropylenimine
PLL	poly-l-lysine
CBS	carbosilane dendrimer
Gn	dendrimer of nth generation
siRNA	small interfering RNA
dsiRNA	Dicer substrate siRNA (siRNA precursor)
miR	synthetic mimics of microRNA
antimiR	microRNA antagonist
PEG	polyethylene glycol
PBMC	peripheral blood mononuclear cell
CPP	cell-penetrating peptide
FA	folic acid
